# Lobular Capillary Hemangioma of the Palate -A Case Report

**DOI:** 10.22038/ijorl.2019.38928.2285

**Published:** 2019-11

**Authors:** Abhay D. Havle, Swapna A. Shedge, Raisha G. Dalvi

**Affiliations:** 1 Department of Otorhinolaryngology, Krishna Institute of Medical Sciences Deemed To Be University, Karad, 415110; Maharashtra, India.; 2 Department of Anatomy, Krishna Institute of Medical Sciences Deemed To Be University, Karad, 415110; Maharashtra, India.

**Keywords:** Diabetes Mellitus, Females, Granuloma-pyogenic, Palate, Tobacco

## Abstract

**Introduction:**

Lobular capillary hemangioma (LCH) is caused by exuberant neovascular response to infection, local irritation (e.g., trauma), or hormonal influence (e.g., pregnancy and consumption of oral contraceptive pills). Pyogenic granuloma is considered to be a misnomer. Although the gingiva is involved in most of the cases, there are is rare cases of extragingival involvement. Herein, we reported a case of LCH associated with the dehiscence of the underlying bony hard palate.

**Case Report:**

A 50-year-old woman presented with a gradually increasing swelling over hard palate for 2 years. She was a hypertensive patient and mishri user (using tobacco-containing teeth cleaning powder) with known diabetes. She had undergone a teeth extraction 2 years ago. The palatine swelling was reddish-blue, sessile with a lobulated surface, firm in consistency, and non-tender with a of size 4×3 cm. The computed tomography (CT) scan revealed bony dehiscence of the underlying palate. Histopathological examination after excision and curettage was suggestive of LCH.

**Conclusion:**

The LCH is common in females due to cyclical hormonal changes. Our case was presented in the fifth decade of life. The etiological factors for the patient could be mishiri usage or iatrogenic trauma of teeth extraction rather than mere hormones. The dehiscence of the underlying palatine process of the maxilla could be due to the acquired invasive nature of the lesion. No recurrence was observed in our patient since the elimination of the lesion and strict abstinence from mishiri till this date.

## Introduction

Lobular capillary hemangioma (LCH) is a common, acquired proliferative reaction of soft tissues. It is neoplastic vascular proliferation which can occur over the skin or mucosa (1). Clinically, these lesions usually present as a single nodule or sessile papule with smooth or lobulated surface with ulceration and bleeding. These lesions may be seen in any size from a few millimeters to several centimeters (2). The clinical appearance is more collagenous and pinkish-blue (2). 

Peak prevalence is in teenagers and young adults with a female predilection of 2:1. The LCH commonly involves the gingiva (75%); however, it may also affect the lips, tongue, buccal mucosa, and palate. These lesions have a higher incidence during pregnancy, which can be due to the elevation of progesterone and estrogen levels (2). Herein, we reported a case of LCH with the dehiscence of the underlying hard palate and intact overlying mucosa. 

## Case Report

A 50-year-old woman presented with right-sided swelling over the hard palate 2 years before the study. She was taking antihypertensive and oral hypoglycemic medicines for hypertension and was recently diagnosed with type II diabetes mellitus. The patient was a mishiri user for 30 years. She underwent the extraction of the right upper 1^st^ and 2^nd^ molars 2 years ago. She developed reddish-blue sessile swelling with a lobulated surface of 4×3 cm that was roughly extended from the premolar to the third molar of the right upper jaw and confined to the hard palate. 

**Fig 1:**
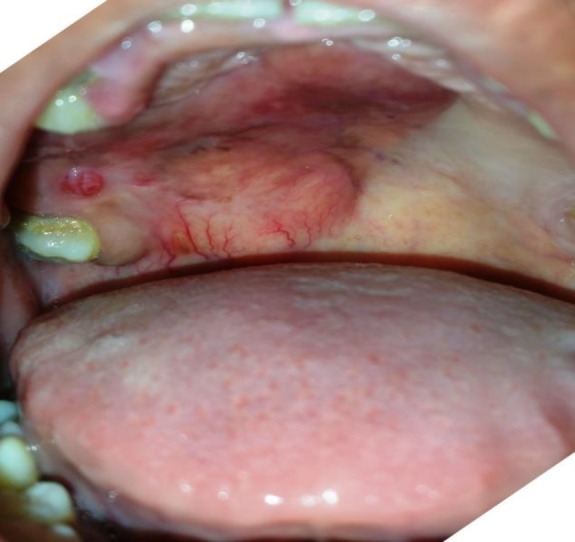
Intra-oral appearance of the lesion

**Fig 2:**
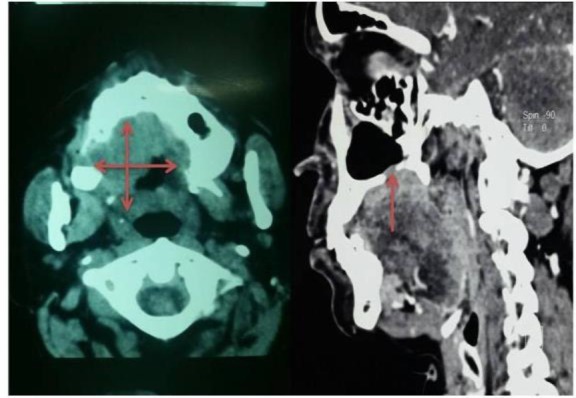
Tomography of the lesion

It was firm in consistency and non-tender with smooth overlying mucosa, limited laterally by the right alveolar margin and medially. It crossed the midline, extended anteriorly about 3 cm from the free margin of the hard palate and posteriorly encroaching the soft palate by crossing the free margin of the hard palate (Fig.1). In addition, the patient had caries of the left upper 3^rd^ molar tooth. The contrast CT scan of the face and neck showed ill-defined soft tissue density along the premolar up to the third molar on the right side and post-contrast enhancement with non-enhancing areas of necrosis within it. The lesion crossed the midline, laterally involved the alveolar process of the maxilla, and extended to the right nasal cavity by eroding through the hard palate (Fig.2). Attempt of fine needle aspiration cytology to rule out the possibility of neoplasia was inconclusive. 

Subsequently, the patient was subjected to subperiosteal excision and curettage. The 2×1 cm bony dehiscence connecting to the right nasal cavity was observed in the right palatine process of the maxilla. The wound was closed primarily. There was a postsurgical oronasal fistula requiring temporary obturator while eating. This fistula eventually was healed by secondary intension. Histopathological examination was suggestive of LCH.

## Discussion

The LCH is inflammatory hyperplasia emerging as a tissue response to irritation, trauma, and hormonal imbalance. This lesion is most commonly seen in the oral cavity and skin (1). It uncommonly occurs extragingivally, in the areas of the lower lip, tongue, and palate, where frequent trauma can occur (2). Hullihen first described a similar case in 1844 (3). The term ‘pyogenic granuloma’ or ‘granuloma pyogenicum’ was coined in 1904 by Hartzell (1). The term, however, is a misnomer as it is neither an infection nor a tumor (1).

Some of the predisposing factors for our case were the constant trauma inflicted by nuts and mishiri, as well as the surgical trauma of dental extraction. In addition, the underlying bone was found to be dehiscent. Conclusive diagnosis is always challenging with such presentations (2). The LCH predominantly occurs in females who are in their second decade of lives (1). However, as observed in our case, it can present in the 5^th^ decade of life. In such conditions, there is an increase in the number of angiogenic growth factors, such as vascular endothelial growth factor and β-Fibroblast growth factor (4). 

The aforementioned growth factors also undergo an increase during pregnancy, therefore, they are ascribed with terms like pregnancy tumor and granuloma gravidarum (4). Two histologic variants of pyogenic granuloma, namely LCH and non-LCH, have been described (5). Histopathologically, pyogenic granuloma is an exuberant vascular proliferation resembling granulation tissue with chronic inflammatory cellular infiltration (1). Microscopically, numerous small and large endothelium lined capillaries engorged with red blood cells are often seen due to endothelial proliferation and formation of numerous vascular spaces (1,5). 

These blood channels are arranged in a lobular pattern therefore termed as “lobular capillary hemangioma”(5). Furthermore, the surface epithelium is atrophic in some areas and hyperplastic in others (1). It is a benign, vascular, reactive tumor of the mucosa or skin (1). 

**Fig 3:**
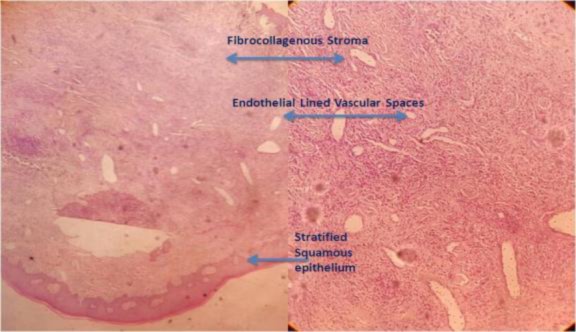
Histopathology of the lesion

Some cases present with multiple pyogenic granuloma known as satellitosis, which may appear as a complication of tumor removal or trauma. Development of multiple satellite lesions may pose difficulties in the diagnosis and management of this condition (1). The histopathological examination of the excision and curettage tissue displayed multiple endothelial lined vascular spaces with fibro-collagenous stroma, which was suggestive of LCH in this case (Fig. 3). 

Various modalities of treatment, such as excision, laser surgery, sclerotherapy, electrodessication, curettage, ligation, or a combination of those, are used in the management of the lesion (1). Excision with linear closure offers the lowest recurrence rate and allows the histological examination of a tissue sample like in our case (1). There is an increased chance of recurrence after surgical excision (2). In our case, the recurrence was not observed 12 months after the excision and curettage. This could be due to the fact that she was a postmenopausal woman and did not indulge into either chewing beetle nut or applying mishiri. However, longer follow-ups may be required.

## Conclusion

Although pyogenic granuloma is common in the second decade of life, it can present in the fifth decade as well in rare conditions. The LCH is more frequently observed in women. Cyclical hormonal changes during reproductive age predispose this clinical condition. Our case presented with LCH at the post-menopausal age of 50 years; therefore, the etiological factors in our patient could be the frequent usage of mishiri rather than mere hormones. In addition, the patient had a history of iatrogenic trauma caused by the right upper teeth extraction 2 years earlier; therefore, this can also be considered as another etiological factor. The dehiscence of the underlying palatine process of the maxilla noted in our case may be due to the acquired invasive lesion. Post-excision recurrence is known; however, in our case with a follow-up of more than 12 months, there were no signs of recurrence. This could be due to the patients’ strict abstinence from mishiri. However, longer follow-up is still essential. 
